# Chronic Exposure to Vinclozolin Induced Fibrosis, Mitochondrial Dysfunction, Oxidative Stress, and Apoptosis in Mice Kidney

**DOI:** 10.3390/ijms231911296

**Published:** 2022-09-25

**Authors:** Davide Di Paola, Ramona D’Amico, Tiziana Genovese, Rosalba Siracusa, Marika Cordaro, Rosalia Crupi, Alessio Filippo Peritore, Enrico Gugliandolo, Livia Interdonato, Daniela Impellizzeri, Roberta Fusco, Salvatore Cuzzocrea, Rosanna Di Paola

**Affiliations:** 1 Department of Chemical, Biological, Pharmaceutical and Environmental Sciences, University of Messina, 98166 Messina, Italy; 2 Department of Biomedical, Dental and Morphological and Functional Imaging, University of Messina, 98125 Messina, Italy; 3 Department of Veterinary Science, University of Messina, 98168 Messina, Italy

**Keywords:** persistent organic pollutants, vinclozolin, kidney impairments, mitochondrial disfunction, apoptosis

## Abstract

Vinclozolin is one of the most used fungicides in the control of fungi in fruits, vegetables, and ornamental plants. The effects of its exposure on different organs have been described, but information regarding its relevance to vinclozolin-induced nephrotoxicity is largely missing. This study focuses on the potential mechanism of vinclozolin-induced nephrotoxicity. CD1 male mice were administered vinclozolin (100 mg/kg) by oral gavage for 28 days. Vinclozolin administration decreased body weight over the treatment period and at the end of the experiment, increased the ratio of kidney weight to body weight and increased serum urea nitrogen and creatinine contents. Vinclozolin also induced histopathological alterations, including tubular dilatation and necrosis and impaired the integrity of the renal-tubular architecture and kidney fibrosis. The analyses conducted showed that vinclozolin administration altered the mRNA levels of mitochondrial function-related proteins (SIRT3, SIRT1, PGC-1α, TFAM, NRF1, VDAC-1, and Cyt c) and oxidative stress (increased lipid peroxidation and decreased total antioxidative capacity, catalase, and superoxide dismutase activities, glutathione levels, and glutathione peroxidase activity) in the kidneys. Furthermore, vinclozolin induced toxicity that altered Nrf2 signalling and the related proteins (HO-1 and NQO-1). Vinclozolin administration also affected both the extrinsic and intrinsic apoptotic pathways, upregulating the expression of proapoptotic factors (Bax, Caspase 3, and FasL) and downregulating antiapoptotic factor (Bcl-2) levels. This study suggests that vinclozolin induced nephrotoxicity by disrupting the transcription of mitochondrial function-related factors, the Nrf2 signalling pathway, and the extrinsic and intrinsic apoptotic pathways.

## 1. Introduction

Persistent organic pollutants (POPs) are ever-present anthropogenic contaminants that may bioaccumulate with adverse impacts on the environment and human health. POP exposure is related to one of the worst health challenges: every year more than three million people die from environmental diseases [1]. Among POPs, herbicides and fungicide have been related to the development of environmental nephrosis and hepatosis [2,3,4,5]. Currently, 11.8% of women and 10.4% of men suffered from chronic kidney disease (CKD) with constantly increasing morbidity and mortality [6,7]. Vinclozolin [3-(3,5-dichlorophenyl)-5-methyl-5-vinyloxazolidine-2,4-dione] is a fungicide widely used in Europe and the United States [8] in the control of *Sclerotinia sclerotiorum, Monilinia* spp., and *Botrytis cinerea* on vegetables, fruits, vines, and ornamental plants [9]. In particular, it is the most frequently fungicide used by the wine industry [10]. Many papers in the literature indicate vinclozolin as an environmental and aquatic pollutant [11,12,13]. Vinclozolin dissipates in the environment by microbial-mediated hydrolysis, soil metabolism, abiotic degradation, and transport by water. It is actually hard to define a specific ingested dose of vinclozolin from fruits or vegetables for animals or people because this really depends on how much and how often contaminated foods are assumed.

The United States Environmental Protection Agency (U.S. EPA) has examined dietary (food and water), non-dietary, and occupational exposure to vinclozolin or its metabolites. In general, fungicides have been shown to circulate through the water and air, and it possible for them to end up on untreated foods after application. Consumers alone cannot easily reduce their exposure because fungicides are not removed from produce that is washed with tap water [14]. A key example of exposure to vinclozolin is through wine grapes, which are considered to account for about 2% of total vinclozolin exposure. It has been determined that people may be exposed to residues of vinclozolin and its metabolites containing the 3,5-dichloroaniline moiety (3,5-DCA) through diet, and thus tolerance limits have been established for each crop. Although vinclozolin is not registered for use by homeowners, it is still possible for people to come into contact with the fungicide and its residues. For example, golfers playing on treated golf courses and families playing on sod that was previously treated may be at risk for exposure. Occupationally, workers can be exposed to vinclozolin while doing activities such as loading and mixing.

Vinclozolin is an antagonist of the androgen receptor [15] and causes nipple retention, reduced anogenital distance, and cleft phallus with hypospadias [16], as well as decreased sperm motility, ultimately leading to spermatogenic apoptosis in experimental animals [17,18]. Additionally, it induces important behavioral alterations in rats: decreased preference for social novelty [19] and anxiety in aged males [20], with induced aversion by potential mates [21]. Vinclozolin causes transgenerational epigenetic alterations, moderate or severe glomerular abnormalities, and various types of tumors [22]. Taken together, these studies highlight the different effects of vinclozolin exposure on different organs. Recently, it has been described that the vinclozolin-exposure-induced toxicity is probably due to the increased oxidative stress that induces an imbalance in cellular homeostasis and the overproduction of chemokines and cytokines [23]. It has been described that vinclozolin administration induces oxidative stress in testis by increasing lipid peroxidation and decreasing the activities of antioxidant enzymes in rats. The reported findings are consistent with a major role for accumulated free radical damage in vinclozolin-exposed rats [24].

Well in line with these results, there is also evidence that relates the mechanism of toxicity induced by POP exposure to the overproduction of reactive oxygen species (ROS) [25]. The imbalance in the physiological antioxidant system involves nuclear factor-erythroid 2-related factor 2 (Nrf2) [26]. The Nrf2 signalling pathway is a crucial regulator that modulates the stimulation and/or constitutive expression of antioxidants and phase II enzymes in response to ROS [27]. ROS have been described as inducers of renal dysfunction [28]. The kidney is a crucial organ in homeostasis, with immunological and vital metabolic functions: it is the second largest body organ, after the heart, having the highest mitochondrial number and oxygen consumption [29]. Mitochondria are energetic organelles whose dysfunction relate to a wide spectrum of diseases such as myopathy, hepatopathy, nephropathy, and reproductive disorders [30,31]. Mitochondrial dysfunction, impaired Nrf2 activity, and decreased expression of its antioxidant and nephroprotective targets have been observed in CKD diseases [32,33].

Unfortunately, information relevant to vinclozolin-induced nephrotoxicity is largely missing. This paper focuses on the kidney disfunction induced by vinclozolin exposure. In particular, CD1 mice were orally administered vinclozolin (100 mg/kg) for 28 days. Thereafter, blood samples and kidney tissues were collected from the mice for further assays to evaluate kidney fibrosis, mitochondrial dysfunction, oxidative stress, and apoptosis.

## 2. Results

### 2.1. Vinclozolin Exposure Induces Kidney Dysfunction

**Figure 1:**
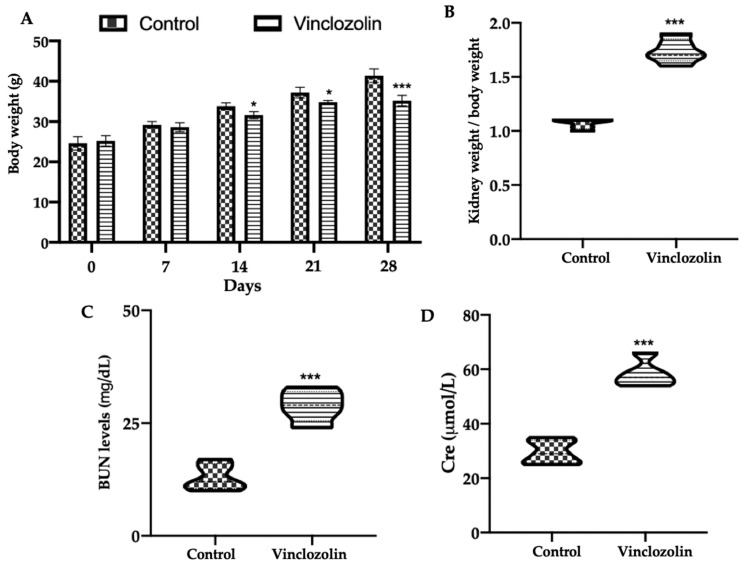
Effects of vinclozolin exposure on kidney function: (**A**) Body weight; (**B**) ratio of kidney weight to body weight; (**C**) BUN content; (**D**) Cre content. A *p*-value less than 0.05 was considered significant. * *p* < 0.05 vs. control, *** *p* < 0.001 vs. control.

The animals’ body weight decreased during the exposure compared to the control (Figure 1A), while the ratio of the kidney weight to the body weight increased after 28 days of vinclozolin (Figure 1B). In order to evaluate the kidney dysfunction, biochemical analyses were conducted. The serum contents of blood urea nitrogen (BUN) (Figure 1C) and creatinine (Cre) (Figure 1D) in the vinclozolin group were prominently higher compared to the control.

### 2.2. Vinclozolin Exposure Induces Kidney Histopathological Alterations

**Figure 2:**
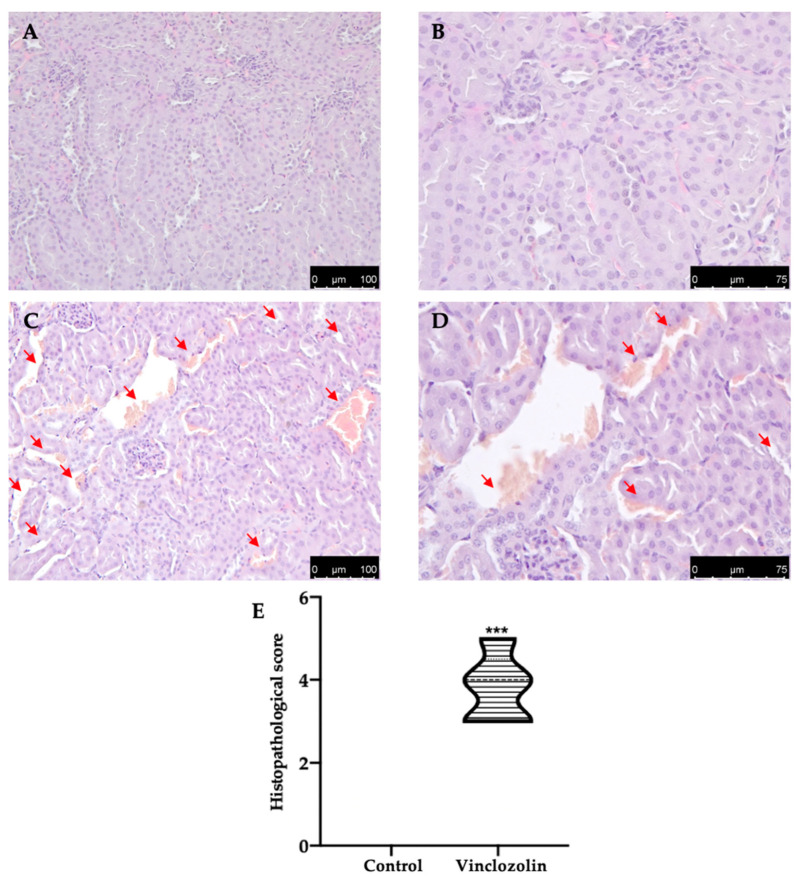
Effects of vinclozolin exposure on kidney histology: H&E staining: (**A**) control magnification 20×; (**B**) control magnification 40×; (**C**) vinclozolin magnification 20×; (**D**) vinclozolin magnification 40×; (**E**) histopathological score. Red arrow: tubular dilatation and necrosis. A *p*-value less than 0.05 was considered significant. *** *p* < 0.001 vs. control.

**Figure 3:**
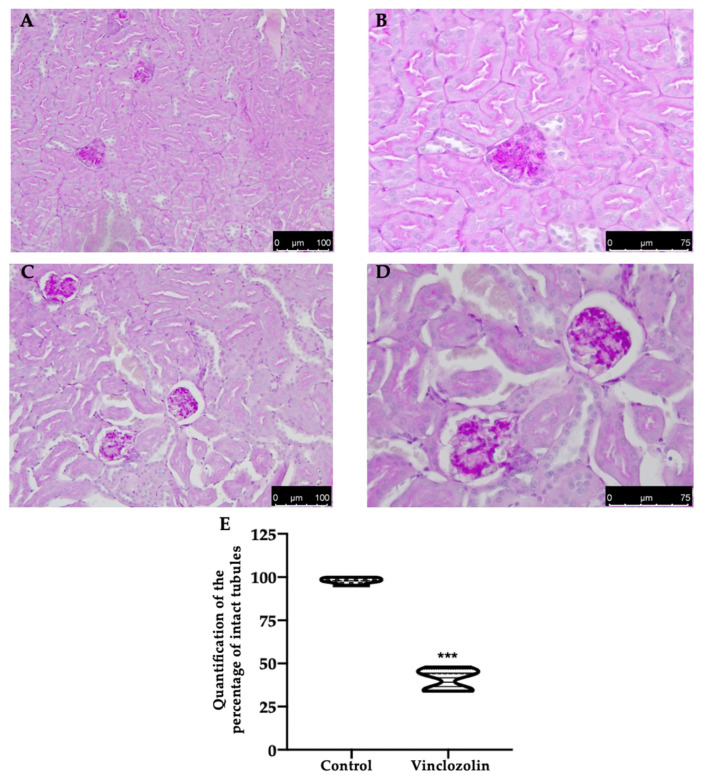
Effects of vinclozolin exposure on kidney histology: PAS staining: (**A**) control magnification 20×; (**B**) control magnification 40×; (**C**) vinclozolin magnification 20×; (**D**) vinclozolin magnification 40×; (**E**) intact tubules %. A *p*-value less than 0.05 was considered significant. *** *p* < 0.001 vs. control.

Vinclozolin exposure induced histopathological alterations, including tubular dilatation and necrosis, as shown by H&E staining (Figure 2C–E) compared to the control (Figure 2A,B,E). PAS staining showed impaired integrity of the renal-tubular architecture in the vinclozolin group (Figure 3C–E) compared to the control (Figure 3A,B,E).

### 2.3. Vinclozolin Exposure Induces Kidney Fibrosis

**Figure 4:**
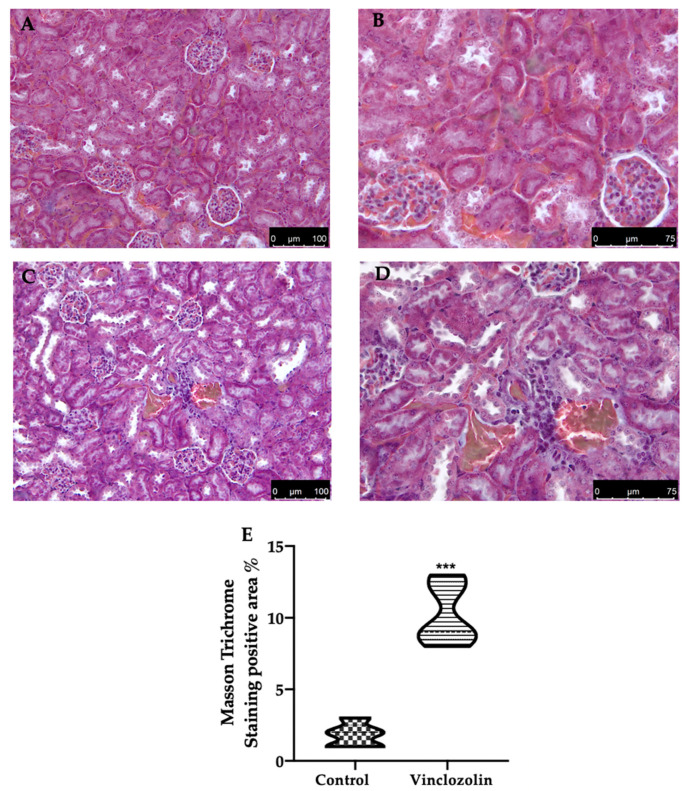
Effects of vinclozolin exposure on kidney fibrosis: Masson Trichrome staining: (**A**) control magnification 20×; (**B**) control magnification 40×; (**C**) vinclozolin magnification 20×; (**D**) vinclozolin magnification 40×; (**E**) Masson Trichrome staining positive area %. A *p*-value less than 0.05 was considered significant. *** *p* < 0.001 vs. control.

**Figure 5:**
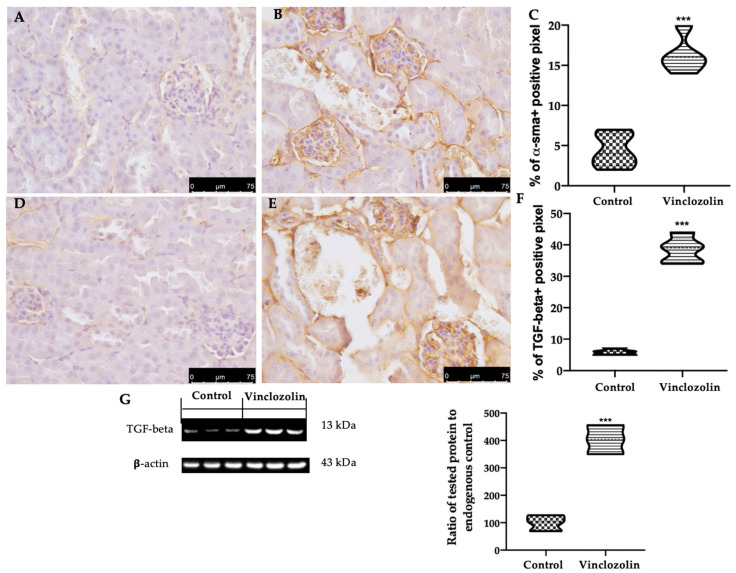
Effects of vinclozolin exposure on kidney fibrosis: Immunohistochemical analysis of α-sma: (**A**) control; (**B**) vinclozolin; (**C**) % of α-sma+ positive pixel, immunohistochemical analysis of TGF-β; (**D**) control; (**E**) vinclozolin; (**F**) % of TGF-β + positive pixel. Western blot analysis of TGF-β expression (**G**). Magnification 40×. A *p*-value less than 0.05 was considered significant. *** *p* < 0.001 vs. control.

To assess the vinclozolin exposure effect on renal fibrosis, Masson Trichrome staining and immunohistochemical analysis were performed. Masson Trichrome staining showed increased collagen deposition in the vinclozolin group (Figure 4C–E) compared to the control (Figure 4A,B,E). Consistently, vinclozolin exposure increased the immunohistochemical expression of α-smooth muscle actin (α-sma) (Figure 5B,C) and TGF-β (Figure 5E,F) compared to the control (Figure 5A,C,D,F). Western blot (Figure 5G) analysis confirmed the increased TGF-β expression in the vinclozolin group.

### 2.4. Vinclozolin Exposure Impaired Mitochondrial Homeostasis in the Kidney

**Figure 6:**
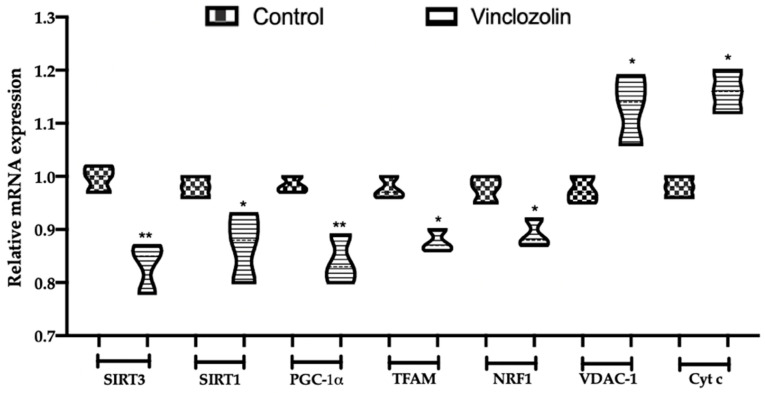
Effects of vinclozolin exposure on mitochondrial disfunction: Relative mRNA levels of mitochondrial function-related proteins. A *p*-value less than 0.05 was considered significant. * *p* < 0.05 vs. control, ** *p* < 0.01 vs. control.

To evaluate the impact of vinclozolin exposure on mitochondrial homeostasis, the transcriptional levels of mitochondrial function-related factors were evaluated. The mRNA levels of SIRT3, SIRT1, PGC-1α, TFAM, and NRF1 were downregulated in the vinclozolin group compared to the control (Figure 6). Conversely, vinclozolin exposure increased the expression of VDAC-1 and Cyt c compared to the control (Figure 6), compromising mitochondrial function in the kidney.

### 2.5. Vinclozolin Exposure Impaired Oxidative Homeostasis in the Kidney

**Figure 7:**
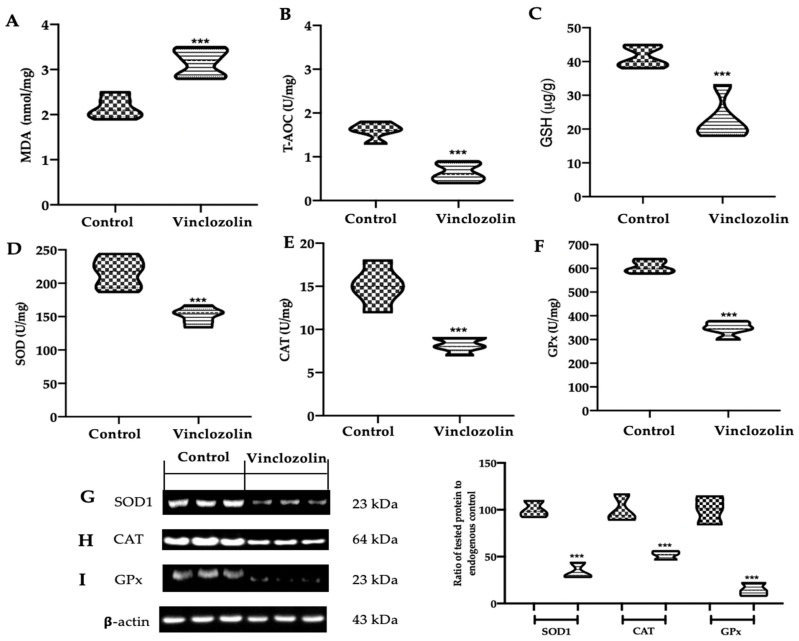
Effects of vinclozolin exposure on oxidative stress response: (**A**) MDA content; (**B**) T-AOC levels; (**C**) GSH levels; (**D**) SOD activity; (**E**) CAT activity; (**F**) GPx activity, Western blot analysis of (**G**) SOD1; (**H**) CAT; and (**I**) GPx expressions. A *p*-value less than 0.05 was considered significant. *** *p* < 0.001 vs. control.

**Figure 8:**
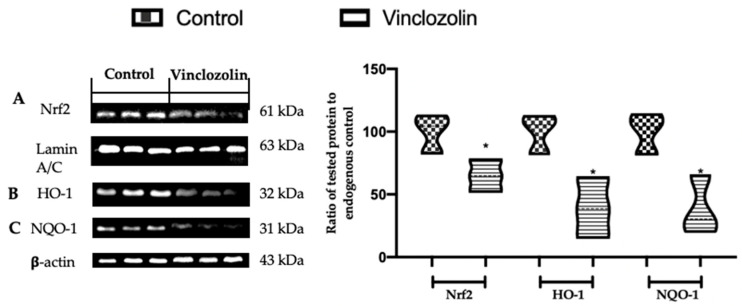
Effects of vinclozolin exposure on the oxidative stress response: Western blot analysis of (**A**) Nrf2; (**B**) HO-1; (**C**) NQO-1 expressions. A *p*-value less than 0.05 was considered significant. * *p* < 0.05 vs. control.

Considering the importance of oxidative damage in mitochondrial dysfunction, the malondialdehyde (MDA) content and total antioxidant capacity (T-AOC) levels were investigated. Vinclozolin exposure significantly increased lipid peroxidation (Figure 7A) compared to the control while the T-AOC levels significantly decreased (Figure 7B). In addition, we measured the GSH, CAT, SOD, CAT, and GPx activities because they have been described as indispensable antioxidants that prevent or resist oxidative damage. Vinclozolin exposure significantly decreased GSH (Figure 7C), SOD (Figure 7D,G), CAT (Figure 7E,H), and GPx (Figure 7F,I) activities compared to the control. The Nrf2 pathway has a key role in in the amelioration of oxidative injury. Western blot analysis showed decreased Nrf2 expression in the nucleus of samples harvested from the vinclozolin group compared to the control (Figure 8A). Well in line with this result, the levels of Nrf2 downstream proteins HO-1 (Figure 8B) and NQO-1 (Figure 8C) in the cytosol were significantly decreased in the vinclozolin group compared to the control.

### 2.6. Vinclozolin Exposure Stimulates Apoptosis Induction in the Kidney

**Figure 9:**
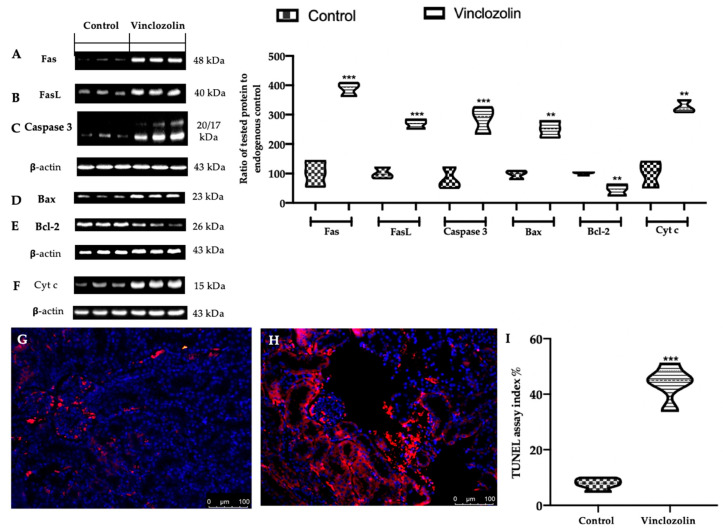
Effects of vinclozolin exposure on apoptosis: Western blot analysis of (**A**) Fas; (**B**) FasL; (**C**) Caspase 3; (**D**) Bax; (**E**) Bcl-2; (**F**) Cyt c expression, TUNEL-staining: (**G**) control; (**H**) vinclozolin; (**I**) TUNEL assay index %. Magnification 20×.A *p*-value less than 0.05 was considered significant. ** *p* < 0.01 vs. control, *** *p* < 0.001 vs. control.

To investigate the apoptosis response, the levels of pro-apoptosis mediators (Fas, FasL, Bax, and Caspase3) and the anti-apoptosis mediator (Bcl-2) were evaluated. Western Blot analysis showed increased Fas (Figure 9A), FasL (Figure 9B), Bax (Figure 9C), and Caspase 3 (Figure 9D) expression in the vinclozolin group compared to the control. Vinclozolin exposure decreased the Bcl-2 (Figure 9E) level compared to the control. Western blot analysis confirmed the increased Cyt c expression in the vinclozolin group (Figure 9F) compared to the control. The vinclozolin group showed an increased TUNEL-assay index % (Figure 9H,I) compared to the control (Figure 9G,I).

## 3. Discussion

Recently, the exposure to environmental pollutants has been widely recognized to cause harm to farm animals and human health [34,35]. It has been shown that 25–33% of diseases are linked to POP exposure. The studies conducted so far showed that prolonged exposure to POPs negatively affected growth, postnatal development, and the endocrine system [16,17,23]. Vinclozolin studies are mainly focused on the reproductive system. It was demonstrated that exposure between the first embryonic day and the first postnatal day resulted in anomalies of the external genitalia in the neonatal stage, including a shortening of the anogenital distance and the retention of nipples in male pups. Due to a cleft phallus and hypospadias, male rats do not perform intromission or ejaculate during maturation. Additionally, males exhibit ectopic testicles, vaginal pouches, epididymal granulomas, accessory sex glands that are smaller or missing, and fewer cauda epididymal sperm cells. Additionally, vinclozolin prepubertal exposure in male rats inhibits pubertal maturation and slows the growth of the accessory sex gland and epididymis [36]. D’Amico and colleagues recently demonstrated that the effect of endocrine disruptor exposure was not limited to the urogenital apparatus [23,37].

Oxidative stress is one of the main investigated mechanisms of POP toxicity [25]. The impaired oxidative homeostasis includes a reduced level of antioxidant enzymes and an increased expression of pro-oxidative mediators, which contribute to organ toxicity [38,39,40]. The overproduction of ROS/oxidative stress impaired kidney function as showed by increased serum BUN and Cre in vinclozolin-exposed animals. The impaired functionality is well in line with the histological modifications. Vinclozolin previously demonstrated the ability to induce histological alteration in the testis, prostate, ovary, kidney, and gonadal fat pad [41]. Our data showed that vinclozolin exposure induced injury in the kidney structure, including vacuolation, hypertrophy, necrosis, and swelling. These features promote the progression of subsequent kidney fibrosis. Masson trichrome staining showed increased collagen deposition, while immunohistochemical analysis showed increased TGF-β and α-sma expression in vinclozolin-exposed animals, which is related to the impaired functionality. These results were also confirmed by Western blot analysis for TGF-β.

The kidney is an active metabolic organ that creates large quantities of adenosine triphosphate via oxidative phosphorylation with an abundant number of mitochondria [42]. Mitochondria are key regulators of cellular homeostasis in metabolism, biosynthesis, apoptosis, and biological monitoring [43]. Mitochondrial dysfunction contributes to nephrosis, including CKD, diabetic nephropathy, and renal function defects [42,44]. Under mild stress conditions, mitochondria exert a protective effect by indirectly or directly upregulating several regulating genes that contribute to mitochondrial homeostasis, including PRX3, SIRT3, SIRT1, PGC-1α, TFAM, and NRF1 [45]. TFAM binds to the mitochondrial genome and controls DNA transcription and replication [46].

SIRT1 triggers the PGC-1α-mediated transcription of mitochondrial and nuclear genes encoding for proteins involved in oxidative phosphorylation, mitochondria proliferation, and energy production, while SIRT3 activates the proteins essential for the tricarboxylic acid cycle, oxidative phosphorylation, and fatty-acid oxidation and indirectly, AMPK and PGC-1α [47,48].

Under chronic stress, the protective effects of mitochondria fail in maintaining cellular homeostasis. Our data showed that chronic vinclozolin exposure slightly decreased the expressions of SIRT3, SIRT1, PGC-1α, TFAM, and NRF1 but increased VDAC-1 and Cyt c levels, compromising mitochondrial function in the kidney. Impaired mitochondria are responsible for an increased production of ROS, which in turn would bring further harm to mitochondria [49]. Radice and colleagues demonstrated that vinclozolin induced lipid peroxidation in the Hep92 cells line [50]. We found that vinclozolin exposure unbalanced the oxidant–antioxidant equilibrium by increasing MDA levels and reducing antioxidant enzyme activities and the T-AOC content. These results indicated that vinclozolin-induced mitochondrial dysfunction is associated with increased oxidative stress.

One of the most important survival and cellular defence pathways to challenge toxicants and oxidative stress is the Nrf2 signalling pathway [51]. Under unstressed conditions, the cytoplasmic inhibitor Keap1 keeps Nrf2 in a quiescent state. Under oxidative conditions, the cytoplasmic complex is wrecked and Nrf2 translocates into the nucleus to induce the transcription of phase II detoxifying and antioxidant enzymes [52]. Our data showed that vinclozolin exposure deactivated the Nrf2 signalling pathway, weakening the activities of antioxidant enzymes, including NQO-1, HO-1, CAT, and SOD.

Increased oxidative and impaired mitochondrial homeostasis induced by vinclozolin exposure relates to increased apoptosis. Apoptosis is a physiological process of controlled cell death [53]. The pathways involved may be divided into intrinsic and extrinsic [54]. Fas/FasL mediate extrinsic signalling, while the intrinsic pathway is regarded as a mitochondrial-mediated event [53]. During apoptosis, many mitochondrial events occur, including the uncoupling of oxidative phosphorylation [55], generation of ROS [56] and Cyt c, and the release of other proteins [57]. Cyt c, upon entering the cytosol, triggers the activation cell death proteases known as caspases [58,59]. Overexpression of Bcl-2 prevents the release of Cyt c and activation of caspases, whereas Bax induces these changes [60]. Different studies demonstrated that vinclozolin administration induces granulosa cell apoptosis during follicular atresia in pigs as well as apoptosis in the testis [61,62]. Vinclozolin exposure increased Fas, FasL, and Bax expressions but reduced Bcl-2 expression. Both extrinsic and intrinsic apoptotic pathways have Caspase 3 as the executer [63]. The increased expression of Caspase 3 in the vinclozolin-exposed group indicated that the two apoptotic pathways were interrelated. The cross-talk between extrinsic and intrinsic signalling pathways would have synergistic effects, aggravating the toxic effect of vinclozolin in the kidney.

## 4. Materials and Methods

### 4.1. Animals

CD1 mice (male 20–22 g; age 6–8 weeks) were purchased from Envigo (Milan, Italy) and employed for this study. The research was authorized by the University of Messina’s Animal Care Review Board (P.R.904/2021). All animal experiments complied with the Italian and international regulations in Italy (D.Lgs 2014/26) and EU regulations (EU Directive 2010/63).

### 4.2. Experimental Groups

Mice were randomly assigned to different groups, as described below:

- Control: mice were orally administered the vehicle for 28 days.
- Vinclozolin: mice were orally administered vinclozolin (100 mg/kg) for 28 days.

At the end of the experiment, whole blood samples and kidney tissues were collected from mice for further assays.

The dose and route of administration were based on previous studies [23,37] and according to a preliminary experiment (Figure S1). Vinclozolin was administered at three doses (30, 100, and 300 mg/kg) for 7 and 14 days. The selected dose (100 mg/kg) was the one that produced a mild injury because the lower dose did not show any significant effect while the higher dose was already significant at 14 days of administration.

### 4.3. Renal Function Analysis and Renal Coefficient Determination

Whole blood was collected and centrifuged at 1200× *g* for 15 min to obtain serum. The BUN and Cre contents were determined using a Urea Assay Kit and Creatinine Assay Kit in serum according to the manufacturer’s instruction (Roche urea/bun detection kit, 04460715190 and Sigma–Aldrich, St Louis, MO, USA, MAK080). Animals were weighed daily, and the ratio of kidney weight to body weight was calculated.

### 4.4. Histological Analysis

Kidney tissues were fixed at room temperature in buffered formaldehyde solution (10% in PBS) for 24 h, dehydrated using a graded series of ethanol, embedded in Paraplast (Sherwood Medical, Mahwah, NJ, USA), and cut into 7-micrometer-thick sections [64]. Sections were deparaffinized with xylene and stained with hematoxylin and eosin [65], Masson trichrome stain [66], and Periodic Acid-Schiff (PAS) stain [67] according to the manufacturer’s protocol (Bio-Optica, Milan, Italy). Sections were evaluated using a Leica DM6 microscope (Leica Microsystems SpA, Milan, Italy) equipped with a motorized stage and associated with Leica LAS X Navigator software (Leica Microsystems SpA, Milan, Italy) [68].

Histopathology scoring was applied as described previously [69,70] in a blind fashion. The score was given based on the grading of tubular necrosis, loss of brush border, cast formation, and tubular dilatation as follows: 0 (none), 1 (≤10%), 2 (11–25%), 3 (26–45%), 4 (46–75%), and 5 (≥76%).

### 4.5. Western Blot Analysis

Western blot analyses were done as previously described [71].

Cytosolic and nuclear extracts were prepared as follows [72]: kidney tissues from mice were suspended in extraction Buffer A containing 0.2 mM PMSF, 0.15 mM pepstatin A, 20 mM leupeptin, and 1 mM sodium orthovanadate, homogenized at the highest setting for 2 min, and centrifuged at 12,000 rpm for 4 min at 4 °C. Supernatants represented the cytosolic fraction. The pellets, containing enriched nuclei, were resuspended in Buffer B containing 1%Triton X-100, 150mMNaCl, 10mM Tris–HCl pH 7.4, 1mMEGTA, 1mMEDTA, 0.2 mM PMSF, 20 mm leupeptin, and 0.2 mM sodium orthovanadate [73]. After centrifugation for 10 min at 12,000 rpm at 4 °C, the supernatants containing the nuclear protein were collected. Protein concentrations were estimated by the Bio-Rad protein assay using bovine serum albumin as a standard. Briefly, samples were heated to 100 °C for 5 min, and equal amounts of protein were separated on 10 % SDS-PAGE gel and transferred to PVDF membrane. Membranes were probed with one of the following primary antibodies: anti-SOD1 (Santa Cruz Biotechnology, Dallas, TX, USA, sc-8637), anti-CAT (Santa Cruz Biotechnology, sc-34280), anti-GPx (Santa Cruz Biotechnology, sc-22146), anti-cyt c (ab133504), anti-TGF-beta (Santa Cruz Biotechnology, sc-130348), anti-Fas (Santa Cruz Biotechnology, sc-74540), anti-FasL (Santa Cruz Biotechnology, sc-19988) or anti-Nrf2 (Santa Cruz Biotechnology, sc-365949), or anti-HO-1 (Santa Cruz Biotechnology, sc-136960), or anti-NQO-1 (Abcam, Boston, MA, USA), anti-Caspase 3 (Santa Cruz Biotechnology, sc-7272), or anti-Bax (Santa Cruz Biotechnology, sc-20067), or anti-Bcl-2 (Santa Cruz Biotechnology, sc-7382) in 1 x PBS, 0.1% Tween-20, 5% w/v non-fat dried milk (PMT) at 4 °C overnight [74]. Membranes were incubated with peroxidase-conjugated bovine anti-mouse IgG secondary antibody or peroxidase-conjugated goat anti-rabbit IgG (1:2000, Jackson ImmunoResearch, West Grove, PA, USA, cat 115-035-174 and 111-035-003) [74]. Blots were also incubated with primary antibody against β-actin protein (1:10,000; Sigma–Aldrich Corp., A5441) or lamin (1:10,000; Sigma–Aldrich Corp., SAB4200236), used as internal standards [75]. Signals were detected with an enhanced chemiluminescence detection system reagent according to the manufacturer’s instructions (SuperSignalWest Pico Chemiluminescent Substrate, Pierce) [76]. The relative expression of the protein bands was quantified by densitometry with Bio-Rad ChemiDocTMXRS+software and standardized to β-actin levels. Images of blot signals (8-bit/600-dpi resolution) were imported into analysis software (Image Quant TL, v2003).

### 4.6. Renal Oxidative Stress and Antioxidant Enzyme Assays

Determination of SOD activity was performed as previously described [71]. GSH levels were determined using a microplate reader at 412 nm and expressed as ng/mg wet tissue [77]. Glutathione peroxidase activity was estimated by measuring the oxidation of guaiacol in the kidney according to a standard method [78]. Lipoperoxidation was estimated using the thiobarbituric acid reactive substances (TBARS) test [79]. MDA levels were expressed as nmol/g wet tissue weight [80]. Determination of CAT activity was performed as previously described [81]. Total antioxidative capacity (T-AOC) levels were measured using a commercially available kit following the manufacturer’s instructions (Sigma–Aldrich, MAK187).

### 4.7. Immunohistochemical Analysis

Immunohistochemical localization of α-sma and TGF-β was performed as previously described [82]. After deparaffinization, endogenous peroxidase was quenched with 0.3% H_2_O_2_ in 60% methanol for 30 min [83]. The sections were permeabilized with 0.1% Triton X-100 in phosphate-buffered saline (PBS) for 20 min [84,85]. Non-specific adsorption was minimized by incubating the section in 2% normal goat serum in phosphate-buffered saline for 20 min. Endogenous biotin or avidin binding sites were blocked by sequential incubation for 15 min with avidin and biotin. The sections were incubated overnight with primary antibodies: anti-α-sma antibody (Santa Cruz Biotechnology, sc-32251) or anti- TGF-β antibody (Santa Cruz Biotechnology, sc-130348). All sections were washed with PBS and then treated as previously reported [86,87].

Samples were washed with PBS and incubated with secondary antibody (Vectastain Elite, PK-6200, Vector Laboratories, Burlingame, CA, USA). Specific labeling was identified with a biotin-conjugated goat anti-rabbit IgG and avidin–biotin peroxidase complex (Vector Laboratories, Burlingame, CA, USA). The stained sections were observed using a Leica (Wetzlar, Germany) DM6 microscope following a typical procedure [88].

### 4.8. Terminal Deoxynucleotidyl Nick-End Labeling (TUNEL) Assay

Kidney tissues were fixed at room temperature in buffered formaldehyde solution (10% in PBS) for 24 h, dehydrated using a graded series of ethanol, embedded in Paraplast (Sherwood Medical, Mahwah, NJ, USA), and cut into 7-mm thick sections. Sections were deparaffinized with xylene, and apoptosis was analyzed by a TUNEL assay using an in situ cell death detection kit (Roche, Basel, Switzerland, 11684795910) [89,90].

### 4.9. RNA Extraction and cDNA Synthesis

To evaluate the mRNA expression of target genes, RNA was extracted using an RNeasy kit (Qiagen, Milan, Italy) for real-time polymerase chain reaction (PCR) analysis. RNA was quantified with a spectrophotometer (NanoDrop Lite; Thermo Fisher Scientific, Wilmington, DE, USA) [91]. An iScript RT-PCR kit (Bio-Rad, Hercules, CA, USA) was used to synthesize first-strand cDNA according to the manufacturer’s recommendations [92].

### 4.10. Real-Time PCR

In total, 1 μL of total cDNA was used to perform real-time PCR analysis with the SYBR Green method on a StepOnePlus Real-Time PCR System (Applied Biosystems, Waltham, MA, USA) [92]. GAPDH was used as an internal control for normalizing the relative expression levels between samples. For each target gene, besides the biological replicates, three technical replicates were performed. Negative controls using RNA as a template were also included in all runs to test for possible genomic DNA contamination of the samples.

### 4.11. Statistical Evaluation

Data are representative of at least three independent experiments and are expressed as the mean ± SEM from N = 20 mice/group. For each analysis, N = 5 animals were employed as follows: for real-time PCR, N = 5 animals were employed; for Western blot analysis, N = 5 animals were employed; for biochemical analysis, N = 5 animals were employed; and for histological and immunohistochemical analyses, N = 5 animals were employed. The results were analyzed by *t*-tests followed by two-tailed calculations. A *p*-value less than 0.05 was considered significant. * *p* < 0.05 vs. control, ** *p* < 0.01 vs. control, *** *p* < 0.001 vs. control.

## 5. Conclusions

Pesticides have been reported to be associated with kidney disease. Few studies have examined the relationship between individual pesticides and kidney dysfunction. Furthermore, previous epidemiologic studies examining kidney function in relation to occupational pesticide use [93,94,95,96] or environmental exposure to pesticides [97,98,99] have had limited information on specific pesticides or factors affecting levels of exposure. Despite the experimental animal evidence suggesting certain pesticides may be associated with kidney dysfunction [100,101,102,103] and the observed associations between certain pesticides and malignant and non-malignant kidney disease, it remains unclear whether exposure to specific pesticides may contribute to early-stage alterations in kidney function that may ultimately lead to clinically manifested kidney damage.

**Figure 10:**
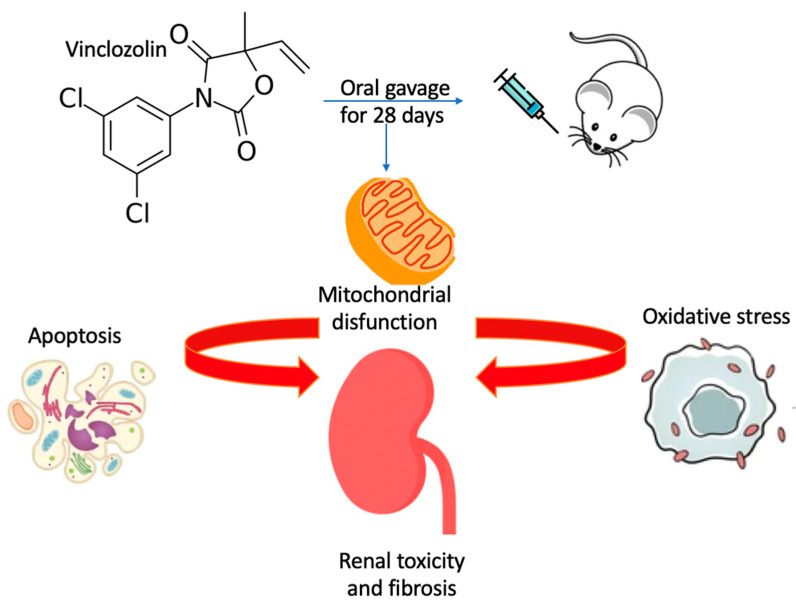
Schematic representation of the proposed signal network.

Overall, this study demonstrated a direct link between renal injury and vinclozolin exposure mediated by impaired mitochondrial homeostasis, increased oxidative stress and apoptosis, organ fibrosis, and altered functionality (Figure 10).

## Data Availability

The data presented in this study are available on request from the corresponding author.

## Supplementary Materials

The following supporting information can be downloaded at https://www.mdpi.com/article/10.3390/ijms231911296/s1.
