# Supplementary material for: Chronic Exposure to Vinclozolin Induced Fibrosis, Mitochondrial Dysfunction, Oxidative Stress, and Apoptosis in Mice Kidney
Source: Int J Mol Sci. 2022 Sep 25;23(19):11296. doi: 10.3390/ijms231911296 (PMC9570110; doi:10.3390/ijms231911296)
Supplement: Supplementary file 1 [file ijms-23-11296-s001.zip › ijms-1872894-supplementary.pdf]

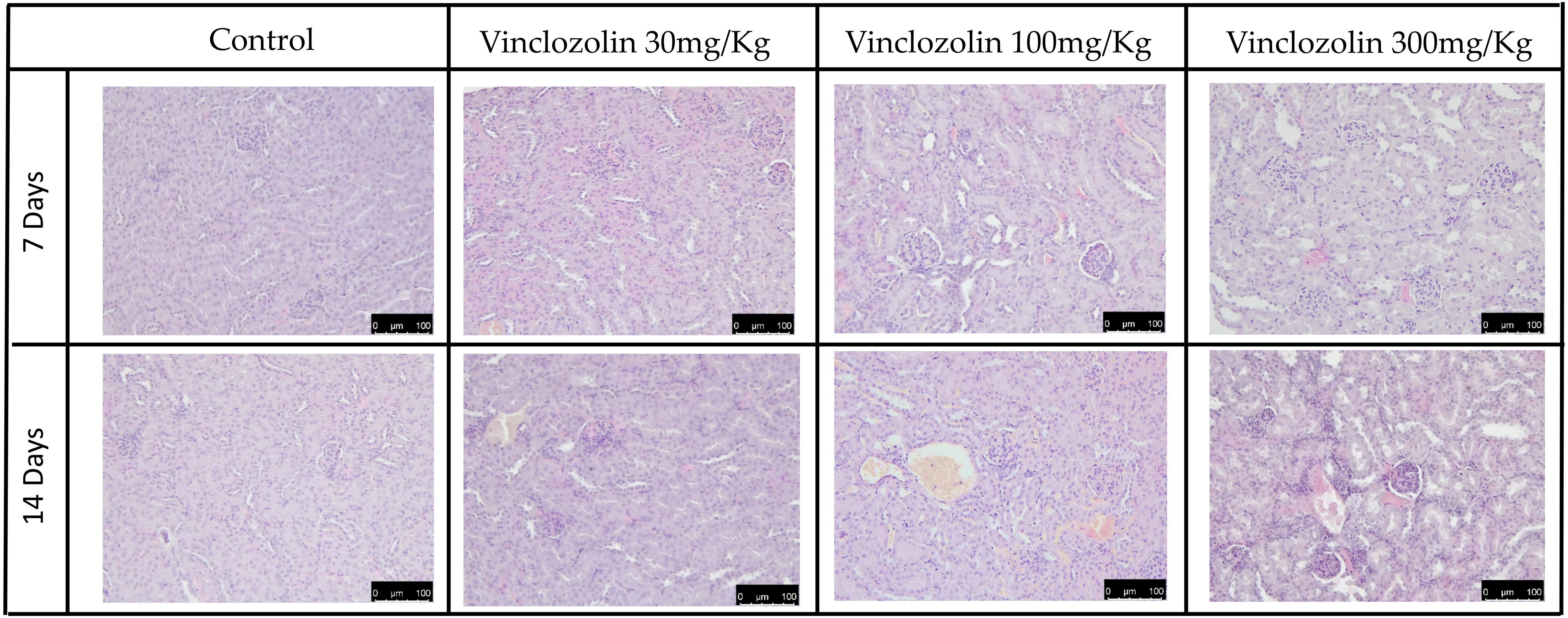

Figure S1: Histological analysis of vinclozolin administration at different doses (30, 100, and 300 mg/kg) for 7 and 14 days
